# Extracellular Vesicle‐Like Nanoparticles Present in Fermented Botanical Products Suppress Fat Absorption in the Gut

**DOI:** 10.1111/1750-3841.70518

**Published:** 2025-08-28

**Authors:** Kotomi Chikama, Koutarou Terada, Chika Yamamoto, Mana Yamamoto, Ayano Hojo, Kotaro Fujioka, Hideto Torii, Lee Wah Lim, Hiroshi Takemori

**Affiliations:** ^1^ Department of Life Science and Chemistry, Graduate School of Natural Science and Technology Gifu University Gifu Japan; ^2^ Department of Research and Development Manda Fermentation Co., Ltd Onomichi Hiroshima Japan; ^3^ Department of Chemistry and Biomolecular Science, Faculty of Engineering Gifu University Gifu Japan

**Keywords:** body weight loss, colon, EVs, fat absorption, fermented food, GLP‐2

## Abstract

**ABSTRACT:**

The fermented botanical product (FBP) is a complex, primarily plant‐based fermented food that has been popular among consumers for many years. Although FBP may modulate gastrointestinal function, the responsible factors and precise mechanisms remain unclear and speculative. Extracellular vesicles (EVs) have gained widespread attention as a novel signaling system, not only in animals but also in plants and microorganisms. Here, we report that FBP contains EV‐like nanoparticles composed of protein complexes originating from various lactic acid bacteria. When mice consumed the EV‐like nanoparticle‐enriched fraction of FBP as a beverage for three weeks, their body weight gain was reduced by up to approximately 10%, particularly in those fed a high‐fat/high‐sucrose (HF/HS) diet. Although the HF/HS diet induced colonic atrophy in mice, the ingestion of the EV‐like nanoparticles‐enriched fraction of FBP increased lipid efflux in feces by more than two‐fold and improved the pathological images of the colon. In vitro experiments on lipase activity suggested lipase inhibitory activity in the EV‐like nanoparticle‐enriched fraction of FBP. Compound analyses revealed that ferulic acid was enriched in the EV‐like nanoparticle fraction and inhibited lipase activity in vitro. The biological effects observed in this study were attenuated by reducing the amount of EV‐like nanoparticles using a polytetrafluoroethylene (PTFE) membrane. However, the slight reduction (not statistically significant) in bioactivity in the PTFE‐treated group compared with the polyether sulfone (PES) treated group, despite a marked decrease in ferulic acid content, suggests the possible involvement of additional molecules beyond EV‐like nanoparticles in this fraction. These findings indicate that FBP may serve as a potential therapeutic option for obesity and gastric symptoms.

**Practical Applications:**

Extracellular vesicle‐like nanoparticles in FBP may support health by alleviating obesity through the suppression of excessive intestinal lipid uptake. These findings suggest that FBP could be developed into a functional food or supplement for individuals who frequently consume high‐fat diets.

## 1 Introduction

EVs, composed of proteins and nucleic acids enclosed within and on the surface of a lipid bilayer, have attracted attention for their diverse physiological roles, influencing both the secreting and recipient cells (Ma et al. 2024; Wu et al. 2024). Not only animal cells but also plants (Sarasati et al. 2023; Suharta et al. 2021) and microorganisms (Arrigan et al. 2024; Li et al. 2024) have been found to secrete or contain EV‐like nanoparticles.

Lactic acid bacteria, such as *Lactobacillus* and *Lactococcus*, are essential symbionts in the human body, especially in the gut and on the skin (Díaz‐Garrido et al. 2021; Nam et al. 2020; Watanabe et al. 2024). They modulate cellular activities, including nutrient digestion and immunity, through intracellular communication (Gwee et al. 2024). To maintain a balanced gut microbiota, consuming fermented foods such as yogurt, cheese, pickles, miso, and vinegar is recommended (Li et al. 2024). EV‐like nanoparticles derived from fermented foods may play a role in mediating communication between microbes and their hosts by modulating host immune and barrier functions (Paula Domínguez Rubio et al. 2017; Melo‐Marques et al. 2024) and contributing to the regulation of gut micro biota balance (Liu 2024).

The fermented foods and beverages mentioned above are typically made from a limited range of ingredients. In contrast, some products, including the present study, undergo fermentation using a diverse source of plant‐based materials and microbial flora. Therefore, we use the term “FBP” to refer to a plant‐based fermented food made from over 53 types of plant‐derived materials, such as cane sugar, along with a variety of fruits, vegetables, grains, and edible algae. It undergoes a fermentation process for over three years at room temperature as a mixture of yeast and lactic acid bacteria derived from the individual materials (Shimada et al. 2004; Yang et al. 2015). The health benefits of FBP have been reported in animal studies, suggesting its regulatory effects on the gut (Fujimura et al. 2018).

Glucagon‐like peptide‐1 (GLP‐1), encoded by the glucagon gene (GCG), is secreted from the ileum and colon after meals and stimulates insulin secretion from the pancreas (Sandoval and D'Alessio 2015). In addition, GLP‐1 has a suppressive effect on appetite, leading to weight loss. Another characteristic of GLP‐1 is its short half‐life, attributed to the digestion by dipeptidyl peptidase 4 (DPP‐4). Consequently, measuring total GLP‐1 levels (both active and inactive forms) in blood is used to predict the potential of GLP‐1. The GCG gene also encodes the GLP‐2 peptide, which supports gut functions, and its receptor agonists are used to treat short bowel syndrome (Pironi et al. 2024), often following partial resection of the intestine after surgical treatment for cancer. In this study, we analyzed EV‐like nanoparticles in FBP using a mouse model, which may provide new insights into fermented foods on gut functions.

## 2 Materials and Methods

### 2.1 Animal Experiments

All experimental procedures were approved by the Animal Committee of Gifu University (2020‐052, AG‐P‐N‐20230002). C57BL/6J mice (seven weeks, males) were purchased from SLC Japan (Shizuoka, Japan). The animals were maintained under a 12 h light/dark cycle (08:00 a.m. – 20:00 p.m.). The mouse diets used in this study, the normal diet CE‐2 and the HF/HS diet D12451 (fat: 45%/cal and sucrose: 17%/cal), were purchased from CLEA Japan (Tokyo, Japan) and Research Diets, Inc. (New Brunswick, NJ, USA), respectively.

To collect feces, mice were transferred to a new cage at 08:00 after the lights were turned on, and feces were collected at 12:00. Blood glucose levels were measured using the Glucocard device (Arkray Inc., Kyoto, Japan) with a drop (1‐2 µL) of blood collected from the tail vein. For the glucose tolerance test, 1 g/kg of glucose was orally administered to mice that had been fasted for 3 h (08:00 a.m. – 11:00 a.m.). When the fasting period exceeded 6 h, most mice consumed their own feces, resulting in significant behavioral artifacts, including body weight loss (Moro and Magnan 2021). To minimize variability in blood glucose levels and ensure a return to baseline within 2 h during the glucose tolerance test in control mice, we adopted a 3‐h fasting in the morning.

Mice were deeply anesthetized with 3% isoflurane (Pfizer Japan, Tokyo, Japan), and blood from the heart and colon samples was collected. Serum GLP‐1 and GLP‐2 levels were measured using enzyme‐linked immunosorbent assay kits (YK161 and YK142, respectively) from Yanahara Co., Ltd., Shizuoka, Japan. Total RNA was purified from the ileal mucosa using the FastGene RNA Premium Kit (Nippon Genetics, Tokyo, Japan), as described by (Mizoguchi et al. 2023). Quantitative polymerase chain reaction (qPCR) was conducted using the reverse transcriptase Revatra ACE and the qPCR enzyme kit Thunderbird SYBR qPCR Mix (TOYOBO Co., Ltd., Osaka, Japan). The specific primers are listed in Supplementary Table 1. qPCR was performed using the MyiQ system (Bio‐Rad), and the ΔΔCt method was employed for data analysis.

The transcriptome (microarray) assay was outsourced to Cosmobio Inc. (Tokyo, Japan) with the Affymetrix Clariom Array (Mouse Clariom S Array: Affymetrix, Santa Clara, CA, USA). Equal amounts of total RNA extracted from the ileal mucosa of four mice in each group were pooled and used for microarray analysis. The web address for the original transcriptome data (Excel file) is provided in Supplementary Table 1.

To measure fecal moisture, the collected feces were dried at 90°C for 6 h, and the weight of the dried feces was measured. The dried feces were rehydrated with 10 volumes of water, and lipid content in feces was measured using the lab assay triglyceride kit (Fujifilm WAKO, Osaka, Japan). Phospholipids and protein in EVs‐like nanoparticles were measured using the lab assay Phospholipid kit (Fujifilm WAKO) and Protein assay (Nacali, Kyoto, Japna). Sectioning and Hematoxylin and Eosin (HE) staining of the ileum were outsourced to BioGate Inc. (Gifu, Japan).

### 2.2 FBP Preparation

FBP was provided by Manda Fermentation Co., Ltd. (Hiroshima, Japan). FBP is produced from 53 or more raw plant indigents, and the approximate composition is 33.4% cane sugar, 26.1% fruits, 14.0% citrus fruits, 8.1% grains, 5.3% root crops, 5.2% pulses, 5.3% edible algae and 2.6% others, fermented and matured with yeasts and lactic acid bacteria for over three years. The matured FBP consists of 60.3% carbohydrate, 32.9% water, 2.6% fiber, 2.2% protein, 1.9% ash, 1% lipid, and several minerals and vitamins (Shimada et al. 2004). Bacteria were detected at less than 300 colony‐forming units per gram, with yeast and *Escherichia coli* being negative (Figure. S1), suggesting a low presence of live bacteria.

Two methods were tested to administer FBP to mice. First, the normal diet CE‐2 powder (CLEA Japan) was mixed with 10% (w/w) FBP (Figure. S2). In the other method, FBP was suspended in 10 volumes of water [10% (w/v)], and the insoluble fraction was removed by centrifugation (8000 rpm for 1 h) with High Speed Refrigerated Centrifuge 6500 (KUBOTA, Tokyo, Japan) and sterilized through a 0.45 µm PES filter (Thermo Fisher Scientific, Waltham, MA, USA), then administered as drinking water (10%). Water with 0.5% dextran was used as the control because the solids content of the 10% water‐soluble fraction of FBP was approximately 0.5%. The feed and drinking water were replaced every two days. As we mention later, the dose of the EV‐like nanoparticles‐enriched fraction was set to 10%, because the nanoparticles fraction was recovered as a 10‐fold concentration (Figure. S2).

### 2.3 Preparation of EV‐Like Nanoparticles‐enriched Fraction

**FIGURE 1:**
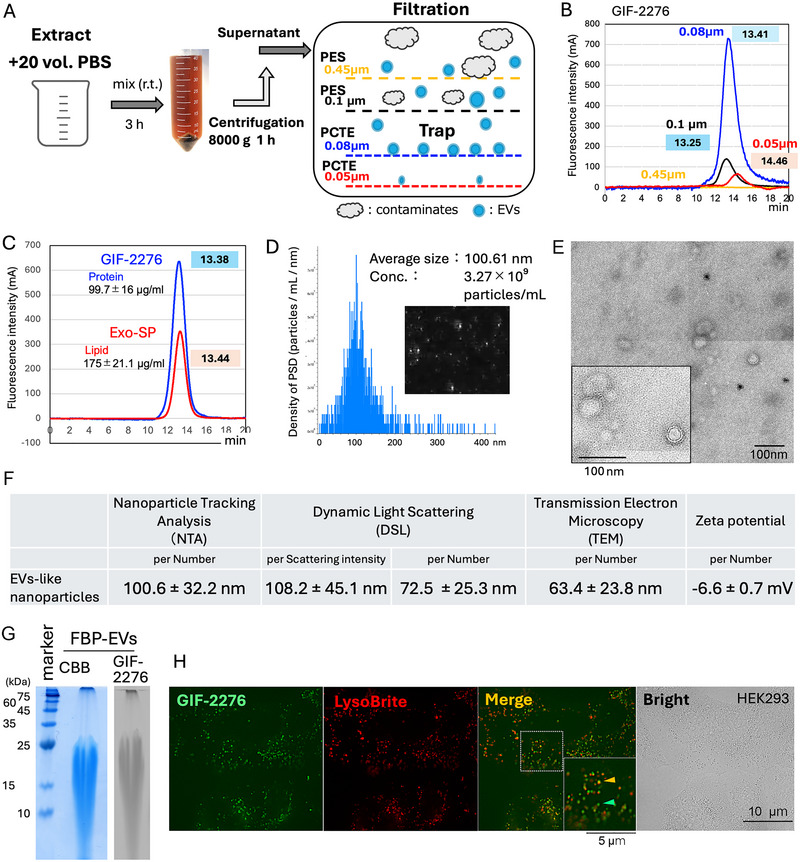
EV‐like nanoparticles in FBP. (A) methods to purify EV‐like nanoparticles; (B) fluorescence‐labeled particles were detected using size exclusion chromatography. The particles were trapped by filters of the indicated sizes, with retention (peak) times shown in boxes; (C) protein (GIF‐2276) and lipid (Exo‐SP) on the particles were co‐labeled and detected simultaneously by two detectors. The concentrations of protein and phospholipid were measured using commercial kits (see materials and methods). *n = 3*; (D) the average size and concentration of the particles were measured using NTA analysis; (E) an image of the particles using TEM analysis; (F) the physical characteristics of EV‐like nanoparticles are summarized. NTA and DSL measurements were performed five times on three independent samples, and TEM analysis was conducted on 50 vesicles; (G) SDS‐PAGE analysis of the GIF‐2276‐labeled particles was conducted. The GIF‐2276‐labeled protein was detected under UV irradiation; and (H) the GIF‐2276‐labeled particles were incorporated into HEK293A cells with pre‐labeled lysosomes in red. In the merged image, colocalized signals appear in orange, while non‐colocalized particles are shown in green.

The water solved fraction of FBP (200mL) was further filtered with a 0.1 µm PES filter (Thermo Fisher Scientific), and the EVs were recovered by polycarbamate track‐etched (PCTE) membrane filter with 0.08 µm pores (GVS Germany GmbH, Sinzig, Germany) (Figure 1A) (Sakurai et al. 2025). The material (particles) trapped on the membrane was washed three times with 5 mL of distilled water and then collected in a final volume of 20 mL (10 times concentrated). The material that passed through the 0.08 µm membrane was subsequently trapped on a 0.05 µm PCTE membrane. EV‐like nanoparticles were labeled with GIF‐2276 and the ExoSparkler Exosome Membrane Labeling Kit‐Red (Exo‐SP) from Dojindo (Kumamoto, Japan), as described in (Furukawa et al. 2024), and separated using an SEC column filled with EV70 resin (GL Science, Tokyo, Japan). To prepare a fraction with a reduced amount of EV‐like nanoparticles, a hydrophilicPTFE membrane with a 0.5 µm pore (Merck Millipore, Burlington, MA, USA) was used instead of a 0.45 µm PES filter.

The average size and concentration of EV‐like nanoparticles were measured by the nanoparticle tracking analyzer (NTA), ViewSizer3000 (Horiba Scientific, Kyoto, Japan). In addition, we performed dynamic light scattering (DLS) analysis using the Zetasizer Nano (Malvern Panalytical, Westborough, MA, USA). The morphological structure of the EV‐like nanoparticles was observed by the transmission electron microscope (TEM) JEM‐2100 (JEOL, Tokyo, Japan).

To analyze the proteins on and within EV‐like nanoparticles, 15% sodium dodecyl sulfate‐polyacrylamide gel electrophoresis (SDS‐PAGE: Fujifilm WAKO) was used, and the protein(s) were identified by the Coomassie brilliant blue staining solution (Kanto Chemical, Tokyo, Japan). Peptide identification by liquid chromatography‐mass spectrometry analysis (LC‐MS) was outsourced to Japan Proteomics, Inc. (Miyagi, Japan).

To perform the cell incorporation assay, HEK293A cells (Thermo Fisher Scientific), maintained in Dulbecco's Modified Eagle Medium (Fujifilm Wako) supplemented with 10% fetal bovine serum (FCS) and penicillin/streptomycin, were used. The concentration of FCS was reduced to 1% for the EV‐incorporation assay. EVs‐like nanoparticles (containing 10 µg of protein) were incubated with 200 µM GIF‐2276 at room temperature for 8 h, and unreacted GIF‐2276 was removed using a 300 kDa PES spin column (Pall Co., Port Washington, NY, USA). The labeled EV‐like nanoparticles were resuspended in 100 µL of PBS, and 10 µL of the suspension was added to the medium of HEK293A cells whose lysosomes had been pre‐labeled with LysoBright Red (ATT Bioquest, Pleasanton, CA, USA).

### 2.4 Lipase Assay

1 µM of 4‐Methylumbelliferyl butyrate, a fluorogenic lipase substrate (Merck), was mixed with 10 µg/mL porcine pancreatic lipase (Nacalai Tesque, Kyoto, Japan) in 200 µL of 10 mM Tris‐HCl (pH 8.0) and incubated at 37°C for 30 min. Then, 150 µL of the reaction mixture was transferred to a 96‐well black plate, and fluorescence was measured using a GloMax‐Multi (Promega, Madison, WI, USA) with a filter set at excitation (Ex) 365 nm and emission (Em) 410–460 nm. The EV‐like nanoparticles‐rich fraction from FBP was added to the lipase reaction mixture after normalization based on lipid content (1 or 5 mg).

Ten‐week‐old male mice (*n = 4*) were fed a normal or HF/HS diet for three days. During the treatment period, drinking water was replaced with PBS (control), an EV‐like nanoparticle fraction obtained by PES filtration, or a fraction obtained by PTFE filtration. On the final day, the mice were subjected to a 3 h fast (8:00 a.m. – 11:00 a.m.) to minimize variation in the amount of undigested food remaining in the gastrointestinal tract, after which the duodenum and cecum were collected. A 5‐cm segment from the proximal duodenum was excised, and its contents were flushed with 0.5 mL of PBS. The flush was diluted 1:100 with PBS for the lipase assay. Feces from the cecum were collected, suspended in an equal volume of PBS, and diluted 1:10 with PBS for the lipase assay.

### 2.5 Compound Assay

Compounds were extracted using equal volumes (1 mL) of methanol from four groups of fractions: before (0.1 µm PES‐filtered) and after recovery of EV‐like nanoparticles. The extracts were concentrated 10‐fold by evaporation, and insoluble materials were removed by centrifugation at 8,000 × g for 30 min. 10 µL of the extract was separated using a C18 octadecylsilica (ODS) column (3.0 × 150 mm, RP‐18 GP; Kanto Chemical, Tokyo, Japan). High‐performance liquid chromatography (HPLC; L‐2200, Hitachi, Tokyo, Japan) and ultra‐performance liquid chromatography (UPLC; Xevo QTof, Waters, Milford, MA, USA) were used, respectively. The solvent gradient (0.4 mL/min) of water and acetonitrile was as follows: acetonitrile 10–100% from 0 to 8 min, maintained at 100% from 8 to 11 min, and returned to 10% from 11 to 15 min for column equilibration. Absorbance at 245 nm was used to monitor the HPLC‐UV separation. For UPLC‐MS detection in negative electrospray ionization mode, the following parameters were set: capillary voltage, 2.0 kV; sampling cone, 31; and extraction cone, 1.0. Authentic ferulic acid was purchased from Tokyokasei, (Tokyo, Japan).

### 2.6 Statistical Analysis

For all experiments, data are expressed as the mean ± standard deviation (S.D.). One‐way analysis of variance (ANOVA) and Student's t‐test were performed using the data analysis tools in Microsoft Excel (WA, USA) for statistical analysis. *, **, and *** indicate *p < 0.05*, *p < 0.01*, and *p < 0.001*, respectively. To evaluate lipase activity, two‐way ANOVA was applied to the two fractions.

## 3 Results

### 3.1 FBP Reduces Weight Gain

To evaluate the potency of FBP in animal models, mice were administered FBP through two distinct feeding methods (Figure S2). In the first method, whole FBP was mixed with powder feed, while in the second method, a part of FBP was provided as a water‐soluble fraction in drinking water. Food consumption could not be accurately measured in the powder feed group due to excessive feed spillage during eating. Therefore, the second method was implemented using solid food and conditioned drinking water groups. The weight ratio of the water‐soluble fraction of FBP (10% w/v) after drying was approximately 0.5% of the water‐soluble FBP fraction. To standardize caloric intake in the drinking water group, 0.5% dextran‐containing water was used as the control. FBP administration via both methods significantly reduced body weight gain (Figure S3A). Monitoring of food consumption suggests that the water‐soluble FBP fraction may reduce food intake (Figure S3B).

GLP‐1 is a potential factor in appetite suppression; therefore, total GLP‐1 levels, including both active and inactive forms, were measured in the serum of these mice. The results showed a trend toward higher GLP‐1 levels in the FBP‐consuming groups, although the differences were not statistically significant (Figure S3C). In addition, no significant differences were observed in the glucose tolerance test (Figure S3D), the condition of the colon, or the fecal status (Figure S3E).

### 3.2 FBP Contains EV‐Like Nanoparticles Derived From Lactic Acid Bacteria

Since the administration of a relatively low dose (10% w/v) of the water‐soluble fraction of FBP significantly reduced body weight gain, we proceeded to identify its components. Considering EVs as a candidate component, we tried to purify EVs or similar substances. The minimal information for studies of extracellular vesicles 2018 (MISEV2018) guidelines recommend ultracentrifugation as the primary method for the isolation and purification of EVs, and filtration methods are also suggested as alternative techniques (Théry et al. 2018). When we attempted ultracentrifugation, fewer particles were precipitated, probably due to the high viscosity of the water‐soluble fraction. Therefore, the particles were purified using a combined filtration method and detected by fluorescent labeling, followed by HPLC‐SEC separation (Figure 1A) (Furukawa et al. 2024, Sakurai et al. 2025). EVs‐like nanoparticles were preferentially captured on the PCTE filter with 0.08 µm pores (Figure 1B). The EVs signals labeled with the protein (GIF‐2276) were almost identical to those labeled with membrane dyes (Exo‐SP) (Figure 1C), suggesting the lipid‐protein complexes.

NTA indicated an average particle size of 100.6 nm with a concentration of 3.3 × 10^9^ particles/mL (Figure 1D), and DLS indicated average particle sizes of 108.2 nm and 72.5 nm, calculated based on scattering intensity and particle number, respectively (Figure S4A). TEM identified particles with lipid bilayer‐like structures (Figure 1E). The zeta potential (membrane surface potential) was measured as −6.6 mV (Figure 1F and S4B). Similar physical properties of EV‐like vesicles have been reported in *Lactobacillus casei* BL23 (Paula Domínguez Rubio et al. 2017). Protein analysis using SDS‐PAGE revealed that GIF‐2276 labeled a protein population with a molecular weight of less than 25 kDa (Figure 1G and Figure S4C), and proteome analyses identified peptides derived from a number of lactic acid bacteria (Table 1), suggesting that the EV‐like nanoparticles in FBP were derived from a complex or consortium of lactic acid bacteria, rather than from a specific species. Interestingly, no peptides derived from vegetables, such as beans, were detected, suggesting that all plant materials might have been completely digested during the three‐year fermentation process. Briefly, the proteome analysis of larger products (> 180 kDa) detected human peptides derived from keratin type 2 (Figure S4D). However, these peptides might have been contaminated during the peptide analysis process. It remains unclear whether the larger products originated from proteins.

**TABLE 1:** Proteome analyses of FBP‐derived EVs Peptides.

Sequence	Candidate protein	Accesion No.	
IMSASGFLLDLVNDVLDMNK	Signal transduction histidine kinase [*Lactobacillus rogosae*]	SFE54024.1	Lactic acid bacteria
EKGPTPNGTLR	Conserved phage C‐terminal domain‐containing protein [*Lactobacillus amylovorus*]	WP_259318193.1	Lactic acid bacteria
VVCIVPHNDPR	Minor capsid protein [*Lactobacillus pasteurii* DSM 23907 = CRBIP 24.76]	KRK07258.1	Lactic acid bacteria
MSATESGTTNKK	C69 family dipeptidase [*Lactobacillus delbrueckii*]	TXJ89480.1	Lactic acid bacteria
VTCDNGDININ	DUF4097 family beta strand repeat‐containing protein [*Lactobacillus bombicola*]	WP_118907219.1	Lactic acid bacteria
ISAKVYGEK	DUF4097 family beta strand repeat‐containing protein [*Lactobacillus* sp. wkB10]	WP_034980933.1	Lactic acid bacteria
IIKIEDLMLK	Hypothetical protein [*Lactobacillus helsingborgensis*]	WP_046327887.1	Lactic acid bacteria
GMGHVLPEALK	Triose‐phosphate isomerase [*Lacticaseibacillus paracasei*]	WP_049181530.1	Lactic acid bacteria
ETSEGIIVEGVDNLLTR	RelA/SpoT family protein [*Limosilactobacillus vaginalis* DSM 5837 = ATCC 49540]	EEJ41285.1	Lactic acid bacteria
TTSLTIAIGMCFLLSR	Hypothetical protein [*Lactobacillus delbrueckii*]	WP_231538363.1	Lactic acid bacteria
EIAAVMAPVTNVNTR	MAG: MerR family DNA‐binding transcriptional regulator [*Lactobacillus sp*.]	RRG09971.1	Lactic acid bacteria
MGLKQADIFNPDGSIK	Integrase [*Lactiplantibacillus plantarum*]	MCG0593542.1	Lactic acid bacteria
GFDEVPPKLAKPMQDR	2, 3‐bisphosphoglycerate‐dependent phosphoglycerate mutase [*Lactobacillus pasteurii*]	WP_009559747.1	Lactic acid bacteria
NLGMTAHYTQSA	Tyrosine‐type recombinase/integrase [*Lactobacillus taiwanensis*]	WP_094517165.1	Lactic acid bacteria
GMMRHLLGQTLTIYK	Ribosome‐binding factor A [*Lactobacillus helveticus*]	WP_259692953.1	Lactic acid bacteria
GFLDEMPNNILTTVK	Gfo/Idh/MocA family oxidoreductase [*Lactobacillus nasalidis*]	WP_201336290.1	Lactic acid bacteria
GMAVMITVASGFRR	Hypothetical protein LACWKB8_0056 [*Lactobacillus* sp. wkB8]	AIS08378.1	Lactic acid bacteria
GFGGLAAASNNPKQAMK	Tape measure protein *[Lactobacillus* sp. PV034]	WP_265488051.1	Lactic acid bacteria
AGLANMMNVMLEISER	Acyl‐ACP thioesterase [*Lacticaseibacillus paracasei* subsp. paracasei Lpp48]	EPD11322.1	Lactic acid bacteria
GHNIMDKLWTSQK	Multispecies: type II CRISPR RNA‐guided endonuclease Cas9 [unclassified *Lactobacillus*]	WP_198180426.1	Lactic acid bacteria
GVPVYTFIAGRAIR	3‐keto‐L‐gulonate‐6‐phosphate decarboxylase UlaD [*Lactobacillus* sp. ESL0260]	WP_122025597.1	Lactic acid bacteria
GMENTNAFGSAIASK	FtsX‐like permease family protein [*Lentilactobacillus diolivorans*]	WP_057865156.1	Lactic acid bacteria
MGVVEIDLHQIK	Galactokinase [Lactobacillus sp. M0396]	WP_198213103.1	Lactic acid bacteria
MGELLKNAWLSLGLSQK	Helix‐turn‐helix transcriptional regulator [*Lactobacillus delbrueckii*]	WP_191669338.1	Lactic acid bacteria
SMIREAAPSGTVAHIVPLK	PTS sugar transporter subunit IIB [*Lactobacillus porci*]	WP_154549327.1	Lactic acid bacteria
LESGDKVELISFVGGG	Sulfur carrier protein ThiS [*Lentilactobacillus hilgardii*]	WP_003553140.1	Lactic acid bacteria
MGFRPGTQVTVDNALK	Conserved protein of unknown function [*Bradyrhizobium vignae*]	SPP98969.1	Soybean rhizobium
KVTDQELWDALAIVQLK	ABC transporter ATP‐binding protein [*Lactobacillus intestinalis*]	WP_057809003.1	Lactic acid bacteria
EYPIAAISTFNPVKR	DUF2252 domain‐containing protein [*Lactobacillus delbrueckii*]	WP_236158061.1	Lactic acid bacteria
GMPVPDVLISGNHQK	tRNA (guanosine(37)‐N1)‐methyltransferase TrmD [*Levilactobacillus namurensis*]	WP_056943702.1	Lactic acid bacteria
GFLTPEDKEDMQLLK	Replication initiation protein [*Lactobacillus* sp. DSM 110155]	WP_251716225.1	Lactic acid bacteria
FGQAIQEVTVAVNSLNAK	Hypothetical protein [*Lactobacillus gigeriorum*]	WP_008473055.1	Lactic acid bacteria
FGVKCINLFEFEK	Hypothetical protein CP353_08000 [*Lactobacillus* sp. UMNPBX2]	PEH09543.1	Lactic acid bacteria
HKFNPEGTTFPMVF	Cytoplasmic protein [*Streptococcus halichoeri*]	WP_159583136.1	Lactic acid bacteria
GFPNSRVLIGLMAS	UbiD family decarboxylase [*Lactobacillus colini*]	WP_209687380.1	Lactic acid bacteria
NHAEASIIRGLNAEAMVK	Hypothetical protein [*Lactobacillus johnsonii*]	MCI6883029.1	Lactic acid bacteria
GIVGLIAPNFGMSAVLLMKFAQEAAK	Dihydrodipicolinate reductase [*Lactobacillus delbrueckii* subsp. lactis]	CDR82589.1	Lactic acid bacteria
FGIPVIADGGIKFSGDIVK	IMP dehydrogenase [*Lachnospira pectinoschiza*]	WP_022501564.1	Lactic acid bacteria
LKFQTAPLK	Xaa‐Pro dipeptidyl‐peptidase [*Lactobacillus* sp.]	HAB49919.1	Lactic acid bacteria
VTPVVTGTTEEV	Cold shock domain‐containing protein [*Lactobacillus* sp. HBUAS51381]	sWP_168899398.1	Lactic acid bacteria
VALTNQTVEQYK	HsdR family type I site‐specific deoxyribonuclease [*Ligilactobacillus ruminis*]	WP_154236478.1	Lactic acid bacteria
QVDIPAMLVATR	Hypothetical protein [*Lacticaseibacillus paracasei*]	WP_016368926.1	Lactic acid bacteria
ILADIAYNDSKTFAQLAETAK	50S ribosomal protein L20 [*Lactobacillus kefiranofaciens*]	WP_250825474.1	Lactic acid bacteria
WP_135960547.1	DNA repair exonuclease [*Lactobacillus intestinalis*]	WP_135960547.1	Lactic acid bacteria
LVLQLNARSLTIYESIPGIAK	IS110 family transposase [*Lactobacillus kefiranofaciens*]	WP_082598976.1	Lactic acid bacteria

Peptide fragments identified by proteome analyses are shown. Database (Accession No.) suggests that these are derived from multiple lactic acid bacteria.

To assess whether EV‐like nanoparticles could be incorporated by cultured cells, GIF‐2276‐labeled EVs were added to the culture medium of HEK293A cells with lysosomes pre‐labeled using LysoBrite Red. After three hours, the EV‐like nanoparticles were incorporated into the HEK293A cells, as evidenced by the co‐localization of EV‐like nanoparticle signals with lysosome signals (Figure 1H). These findings suggest that EV‐like nanoparticles from FBP may undergo degradation in lysosomes after incorporation into mammalian cells.

### 3.3 EV‐Like Nanoparticle‐Enriched Fraction Reduced Body Weight Gain

**FIGURE 2:**
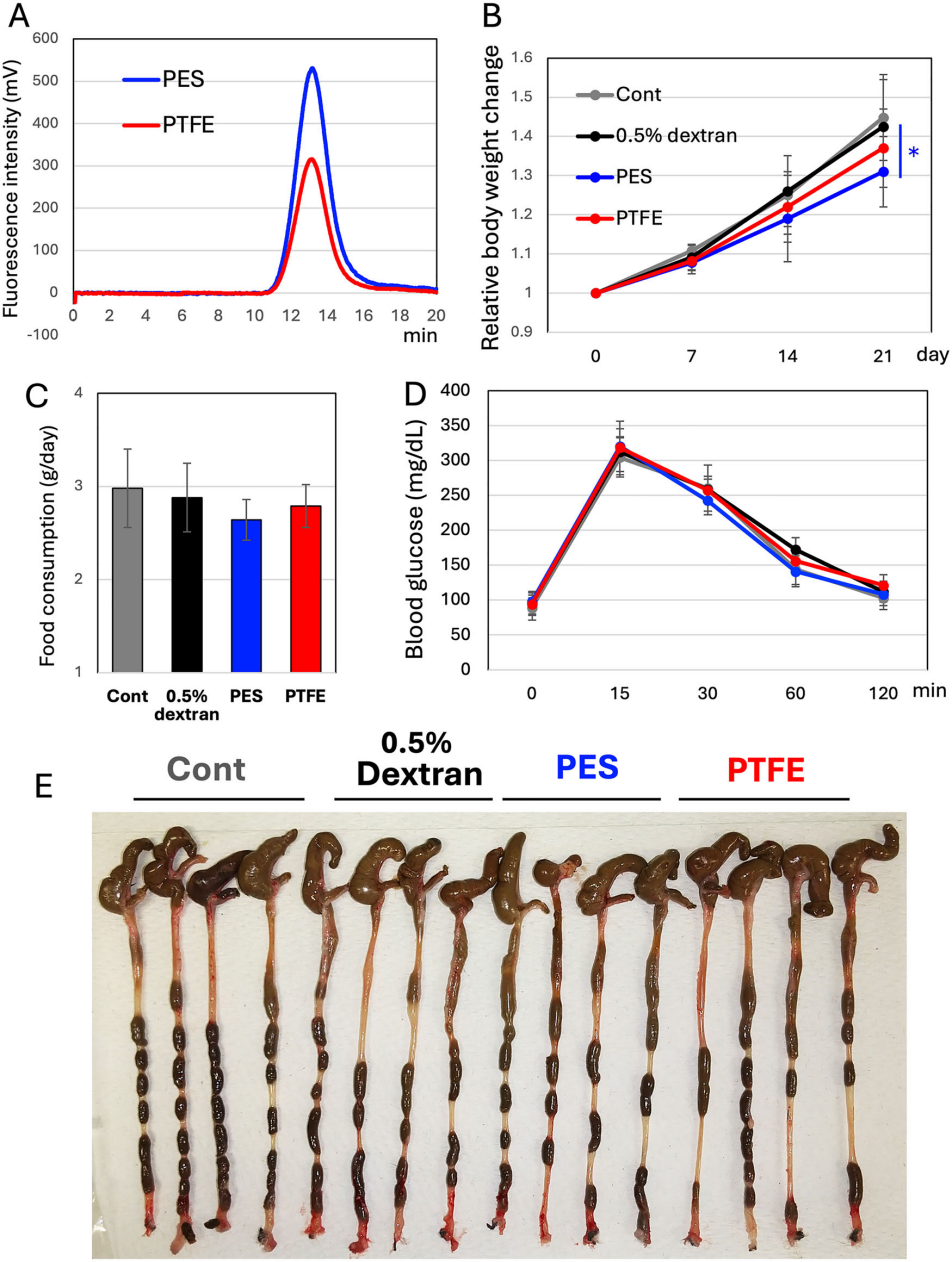
Effects of the EV‐like nanoparticle‐enriched fraction of FBP in mice. (A) EV‐like nanoparticle fractions were re‐filtered using PES or PTFE filters. PTFE filtration resulted in a reduced recovery of EV‐like nanoparticles; (B) EV‐like nanoparticle‐enriched fractions (with a volume/dose equivalent to the water‐soluble fraction of FBP; see Figure S2) were provided to mice as drinking water. Body weight change is expressed as a relative change, with day 0 set to 1. For the controls, normal water (Cont) and 0.5% dextran‐containing water was served. *n = 8*. Means and S.D. are shown. ^＊^: *p < 0.05* (PES group vs. control or 0.5% dextran groups); (C) Food consumption was monitored from the 14^th^ to the 16^th^ day; (D) On the 20^th^ day, mice underwent a 3 h fasting followed by a glucose tolerance test (1 g/kg: oral administration). One to two µL of blood were collected via the tail vein. No significant differences were observed among the groups; and (E) Images of the colons were shown.

Next, we evaluated the biological activities of the EV‐like nanoparticles‐enriched fraction of FBP instead of its water‐soluble fraction. During the filter‐mediated sterilization process, we observed that the use of a PTFE filter (0.5 µm) resulted in a lower recovery of EV‐like nanoparticles compared to a PES filter (0.45 µm) (Figure 2A). Therefore, we considered that PTFE filtration might be useful for preparing semi‐vehicle control groups corresponding to the EV‐like nanoparticle‐enriched fraction, which was prepared using standard PES filters. The EV‐like nanoparticle‐enriched fractions were sterilized (re‐filtrated) through either PES or PTFE filters, and the effects of these fractions were compared. Although the impact on body weight change was smaller compared to the water‐soluble fraction of FBP (Figure S1A), the EV‐enriched fraction prepared through PES filters significantly reduced body weight gain (Figure 2B). While the difference was not statistically significant, a slight reduction in food consumption was noted in the PES filtration group (Figure 2C). No significant differences were observed in the glucose tolerance test (Figure 2D) or in the appearance of the colon (Figure 2E) among all groups. The HE‐staining of the colons also showed no difference among the treatments (Figure S5). Since these experiments on mice were conducted using normal diets, we decided to switch to a diet that promotes an obese condition

### 3.4 EV‐Like Nanoparticle‐Enriched Fraction Efficiently Reduced Body Weight Gain Under a HF/HS Feeding Condition

**FIGURE 3:**
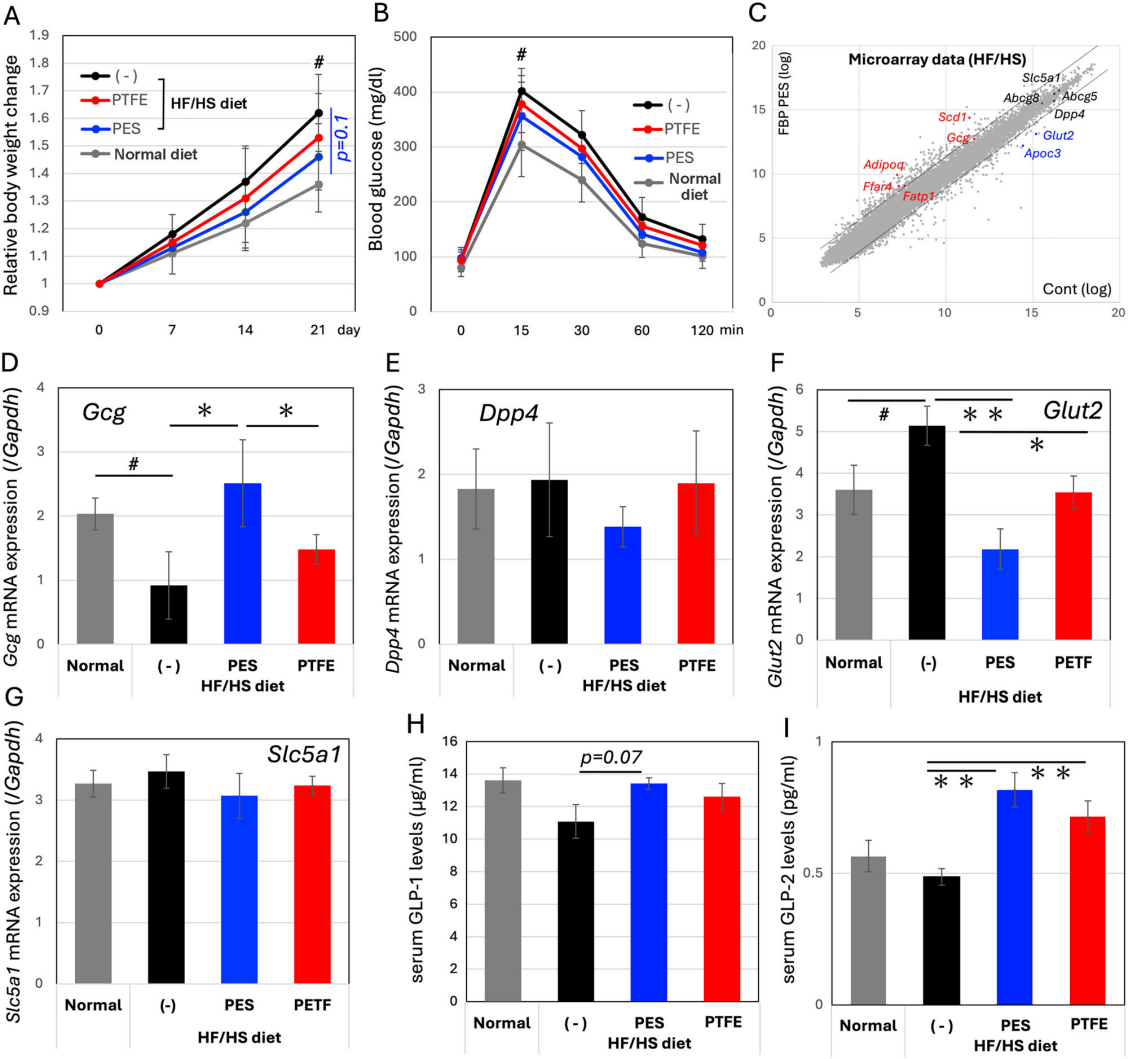
Effects of the EV‐like nanoparticle‐enriched fraction of FBP in mice fed with HF/HS diets. (A) EV‐enriched fractions prepared by PES filtration or PTFE filtration (Figure S2) were provided as drinking water for 21 days. The mice group fed with the HF/HS diet alone served as a negative control for the group that received the PES‐filtrated EV fraction under the HF/HS‐diet feeding. The PTFE‐filtrated EV group served as a semi‐control for the PES group. The normal diet group served as a reference for the HF/HS group. Gray and black lines represent mouse groups fed with a normal diet and a HF/HS diet, respectively, with normal water. Mice weight was monitored every week. *n = 8*. Means and S.D. are shown. ^#^: *p < 0.05* between the normal diet and HF/HS diet groups on 21th day. *p < 0.1* between the control and PES groups fed a HF/HS diet on day 21; (B) on the 20^th^ day, mice underwent a 3 h fasting (08:00 a.m. – 11:00 a.m.) followed by a glucose tolerance test (1 g/kg: oral administration). One to two µL of blood were collected via the tail vein. ^#^: *p < 0.05* between the normal diet and HF/HS diet groups at 15 min after glucose administration; (C) Microarray analysis of intestinal mucosa RNA prepared from mice fed a HF/HS diet vs. those treated with EV‐like nanoparticles by PES filtration. Gene names with abbreviations indicate those related to the absorption of lipids or carbohydrates. Those indicated in red, blue, or black letters represent up‐regulated, down‐regulated, or unchanged genes, respectively. Dotted lines indicate the thresholds for two‐fold changes. **: *p < 0.01*. Quantitative PCR analysis for glucagon (D), DPP4 (E), GLUT2 (F), and SLC5A1 (SGLT1, G) was performed with total RNA prepared from the ileum. *n=8*. The mRNA levels of genes of interest were normalized to those of GAPDH. Serum GLP‐1 (H) and GLP‐2 (I) levels were measured at 12:00 p.m. on the final day. *n = 8*.

When mice were fed a HF/HS diet, the EV‐like nanoparticles‐enriched fraction that prepared through the PES filter reduced body weight gain (Figure 3A) and partially improved glucose tolerance (Figure 3B). Due to the softness of the HF/HS diet, accurate monitoring of food consumption was difficult.

To evaluate the impact of EV‐like nanoparticles derived from FBP on dietary metabolism, we conducted a transcriptome assay. Total RNA was extracted from the ileal mucosa of mice fed the HF/HS diet (control) or supplemented with the EV‐like nanoparticle‐enriched fraction using the PES filter (FBP‐PES group) (Figure 3C). Glucose transporter 2 (Glut2) mRNA levels increased in the FBP‐PES group, while sodium‐glucose cotransporter 1 (SGLT1/SLC5A1) remained unchanged. A similar dissociation in the regulation of these transporters was reported in a study using an anthocyanin‐rich berry extract (Alzaid et al. 2013). The expression of GCG, which encodes glucagon, GLP‐1, and GLP‐2, was up regulated (approximately twofold), whereas the expression of DPP4, which degrades GLP‐1 and GLP‐2, remained unchanged. Since GLP‐1 function are different from that of GLP‐2, DPP4 gene expression may be dissociated from GCG gene expression (Drucker et al. 1997).

Several genes involved in lipid metabolism, SCD1 (stearoyl‐CoA desaturase 1), Adipoq (adiponectin), FATP1 (fatty acid transport protein 1/SLC27A1), and FFAR4 (free fatty acid receptor 4/GPR120, G‐protein coupled receptor 120), were up regulated, whereas APOC3 (apolipoprotein C3) was down regulated. In contrast, ABCG5/ABCG8 mRNA expression, whose products facilitate biliary and dietary sterol efflux from the brush border membrane, remained unchanged.

To precisely analyze these changes, particularly in glucose metabolism, mRNA levels were measured by qPCR (Figure 3D‐G). Because the mRNA levels of glyceraldehyde‐3‐phosphate dehydrogenase (GAPDH) were unchanged among the experimental groups, it was used as an internal control for normalization of target mRNA expression levels (Figure S6). Notably, HF/HS diet feeding reduced GCG mRNA levels, while supplementation with an EV‐like nanoparticles‐enriched fraction prepared via PES filtration restored them beyond levels observed with normal diet feeding. However, this recovery was not evident in the group that received the PTFE‐filtered fraction. GLUT2 mRNA expression was up regulated by HF/HS diet feeding but down regulated by supplementation with the PES‐filtered EV‐like nanoparticles fraction. DPP4 and SLC5A1 mRNA levels remained unchanged across treatments.

To identify factors associated with the changes in GCG mRNA levels, serum GLP‐1 and GLP‐2 levels were measured. GLP‐1 levels were slightly reduced in mice fed the HF/HS diet. However, supplementation with the EV‐like nanoparticle‐enriched fraction prepared via PES filtration tended to restore them (Figure 3H). Unexpectedly, serum GLP‐2 levels increased in groups receiving EV‐enriched fractions prepared via both PES and PTFE filtration (Figure 3I), with a greater increase in the PES group than in the PTFE group. These results suggest that the suppressed expression of GLUT2 mRNA correlates with blood glucose levels.

### 3.5 EV‐Like Nanoparticle‐enriched Fraction Modulates Lipid Metabolism

**FIGURE 4:**
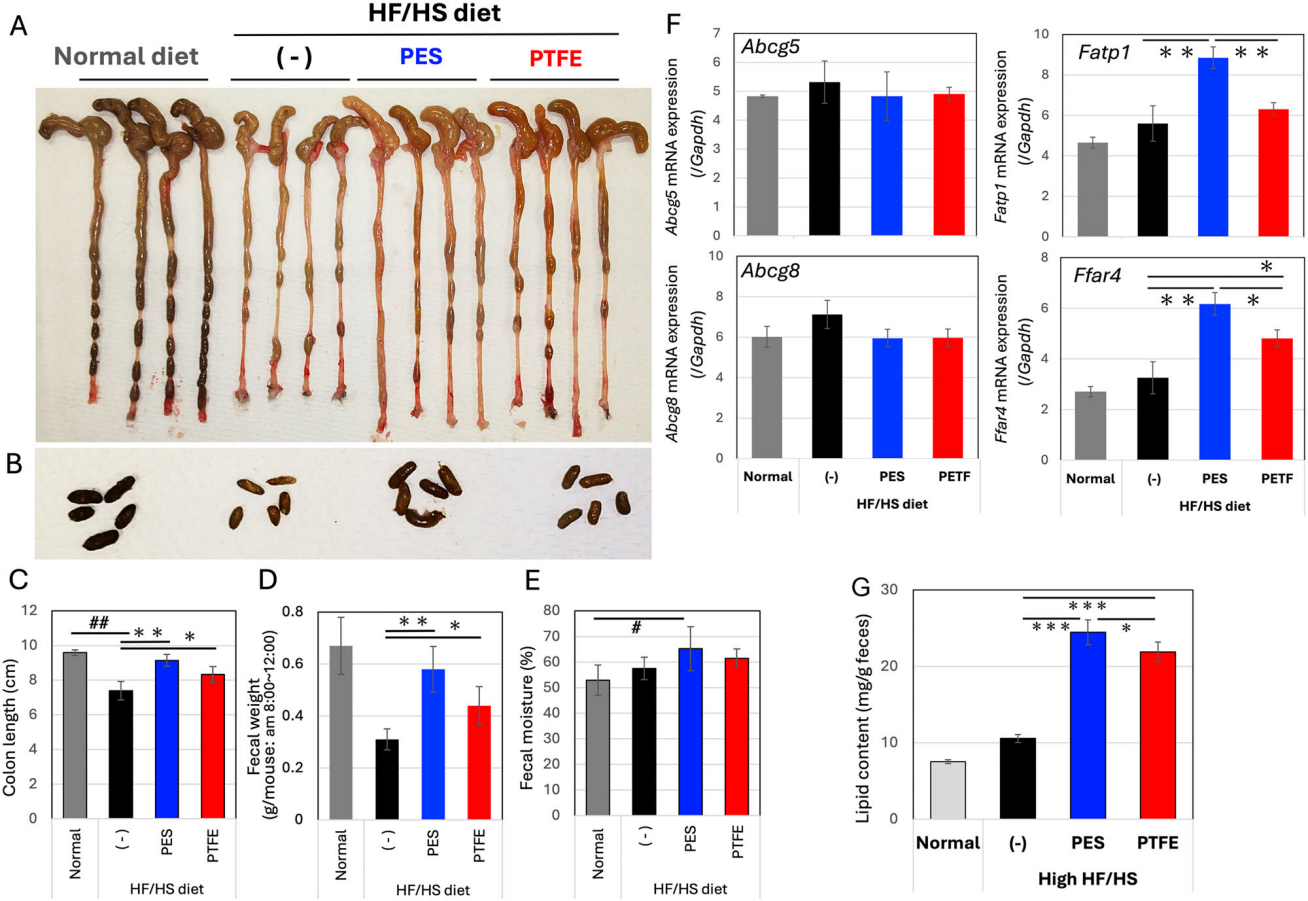
Effects of the EV‐like nanoparticle‐enriched fraction of FBP on gene expression in mice guts. Images of the colons (A) and feces (B) were recorded. C. Colon length were measured. *n = 8*. Means and S.D. are shown. *: *p < 0.05*, **: *p < 0.01*. (^#^ *p < 0.05* between different diet groups.) D. Fecal samples collected at the indicated intervals were measured on the 20^th^ day. *n=4*. E. The feces were dried and reweighed to measure fecal moisture (%). F. Quantitative PCR analysis of ABCG5, ABCG8, FATP1 (SLC27A1), and FFAR4 (GPR120) was performed according to Figure 3. G. The dried feces were rehydrated with 10 volumes of water, and lipid content was measured. *n = 8*, Means and S.D. are shown. ***: *p < 0.001*.

Colon appearance (Figure 4A) and feces (Figure 4B) were also interesting. As reported by other groups (Xie et al. 2020), the colon tended to atrophy in the HF/HS diet group, despite no observable differences in HE staining of the colons among the treatments (Figure S7). The atrophy induced by the HF/HS diet was alleviated by consumption of the EV‐like nanoparticles‐enriched fraction (Figure 4C). Additionally, fecal weight (Figure 4D) and moisture (Figure 4E) were increased in the group that consumed the EV‐like nanoparticles‐enriched fraction.

Consistent with results of the transcriptome assay, ABCG5/8 mRNA levels were unaffected by HF/HS feeding or supplementation with EV‐enriched fractions (Figure 4F). The transporter FATP1 positively regulates the uptake of long‐chain fatty acids in the intestine (Wu et al. 2006), and the receptor FFAR4 is associated with an obese phenotype (Oh et al. 2010; Yasuda et al. 2023). The mRNA levels of both genes were cooperatively up regulated in the groups of EV‐like nanoparticles‐enriched fraction, despite increase in fecal weight (Figure 4B and C). These results suggested that the up regulation of FATP1 and FFAR4 mRNAs was a compensatory response to abnormal lipid absorption in the intestinal tract.

A fecal lipid measurement indicated a significant increase in fecal lipid content in mice groups that consumed the EV‐like nanoparticle‐enriched fractions, particularly in the PES‐filtered group (Figure 4G).

### 3.6 Ferulic Acid in FBP Accumulates in EV‐Like Nanoparticles and Inhibits Lipase Activity

**FIGURE 5:**
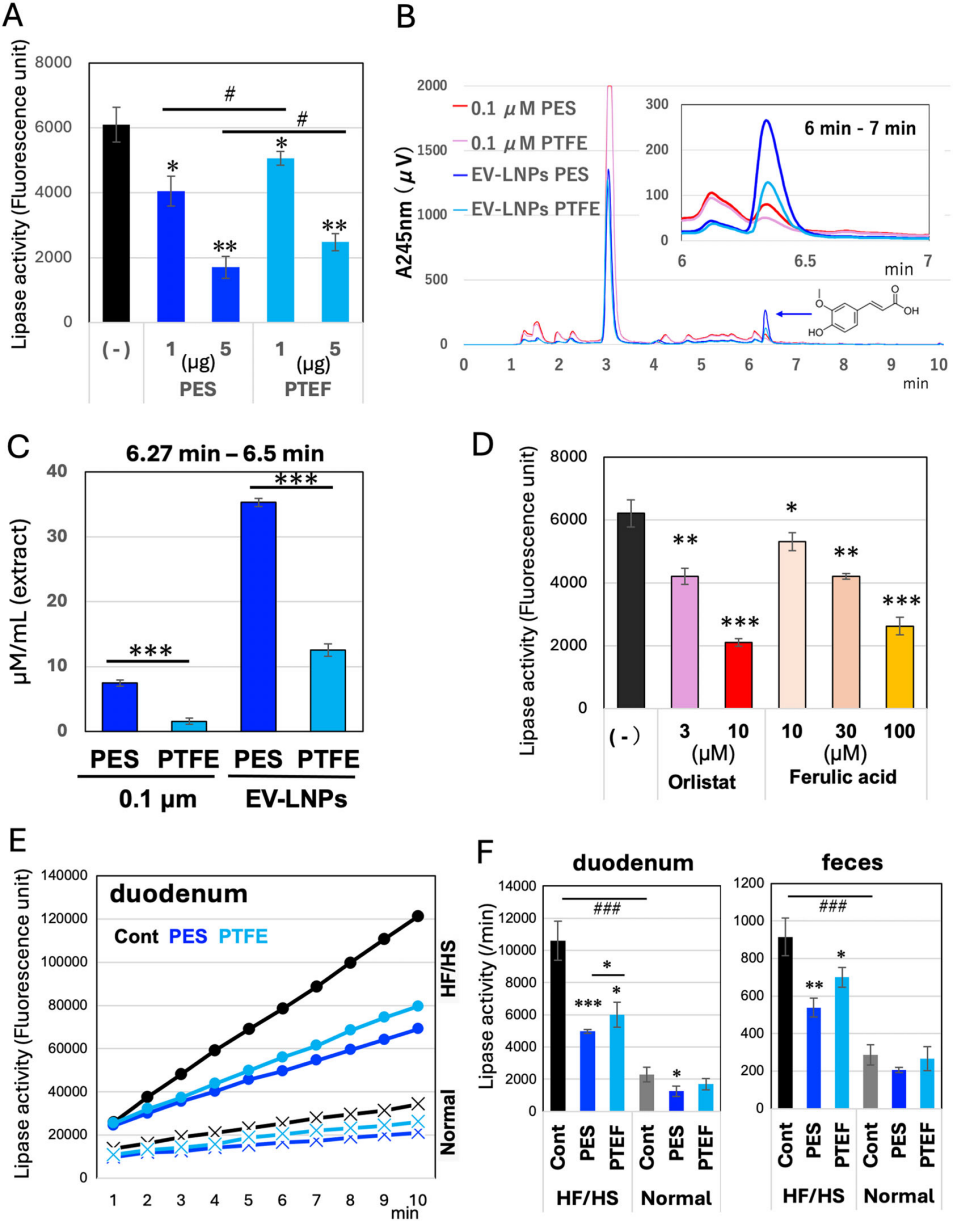
EVs‐enriched fraction of FBP containing ferulic acid and inhibits pancreatic lipase activity. (A) quantification of the inhibitory activity of pancreatic lipase by EV‐like nanoparticle‐enriched fractions (1 or 5 µg lipids) using 4‐methylumbelliferyl as the fluorescent substrate (Figure S8A), *n = 3*. Two‐way ANOVA was used for statistical evaluation. (^#^: *p < 0.05* between different groups.) *p < 0.05, **p < 0.01 compared to control; (B) HPLC‐C18 separation was performed on methanol extracts of each fraction. EV‐like nanoparticles before (0.1 µm PES‐filtered) and after recovery (EV‐LNPs) (Figure 1A) were compared based on their absorbance at 245 nm across four fractions pretreated with either a 0.45 µm PES filter or a 0.5 µm PTFE filter. The chromatogram corresponding to the 6–7 min range is magnified and displayed in the upper right corner; (C) the predicted concentration in the original fraction was estimated from the 6.3 min peak area, assuming that the target compound is ferulic acid (Figure S8C) (Authentic ferulic acid also exhibited a peak at 6.3 min). *n = 8*, Means and S.D. are shown. ***: *p < 0.001*; (D) The inhibitory activities of ferulic acid and the positive control, Orlistat, against pancreatic lipase were measured. *n = 3*. Means and S.D. are shown. *p < 0.05, **p < 0.01 compared to the control (no inhibitor, (–)); (E) comparison of lipase activity in duodenal lumen fluids prepared from six groups of mice. Mice were fed a normal diet or an HF/HS diet together with PBS (Cont), EV‐LNPs with PES filtration or EV‐LNPs with PTFE filtration as substitutes for drinking water. Mean values, corrected for the dilution factor, are plotted (*n = 4*); and (F) lipase activities of the six mouse groups are expressed as fluorescence units per minute. Two‐way ANOVA was used for statistical evaluation (###: *p < 0.001* between different diet groups). * indicates significance compared with the control or between different EV‐LNPs groups. Lipase activities in cecal contents (Figure S8D) are also shown.

The enhanced fecal lipid excretion observed following the consumption of EV‐like nanoparticle‐enriched fractions prompted us to evaluate lipid digestion. An in vitro assay of porcine pancreatic lipase with a fluorogenic substrate (Figure S8A) suggested the presence of lipase inhibitory activity in EV‐like nanoparticle‐enriched fractions (Figure 5A). The fraction containing fewer EV‐like nanoparticles (after PTFE filtration) showed lower lipase inhibitory activity, which was negatively correlated with levels of fecal lipid excretion (Figure 4G). The EV‐like nanoparticle‐enriched fractions from the three lots of FBP exhibited nearly identical inhibitory activity (Figure S8B). These results suggest that FBP may inhibit lipid digestion in the gut, possibly through the action of EV‐like nanoparticles derived from combined lactic acid bacteria (Table 1).

Next, we attempted to identify the factors responsible for lipase inhibition in EV‐like nanoparticle‐enriched fractions. The methanol‐extracted fraction of the water extract of FBP (filtered through a 0.45 µm PES filter or a 0.5 µm PTFE filter followed by a 0.1 µm PES filtration: Figure 1A), as well as the EV‐like nanoparticle‐enriched fractions, were subjected to C18 column chromatography (Figure 5B). Although a prominent peak was detected at 3.2 min, its intensity was reduced in the EV‐like nanoparticle‐enriched fractions. In contrast, the peak at 6.3 min was elevated in the EV‐like nanoparticle‐enriched fractions but was reduced by pre‐treatment with PTFE filtration. Mass spectrometric analysis suggested that ferulic acid might be a component of the 6.3 min peak (Figure S8C).

Assuming that the peak corresponds to ferulic acid, the C18 separation peaks are shown as concentrations in each fraction (Figure 5C). Since ferulic acid has been reported as a potent lipase inhibitor (Salau et al. 2021), we evaluated its lipase inhibitory activity (Figure 5D). Authentic ferulic acid exhibited lipase inhibition, although its activity was weaker than that of the positive control, Orlistat.

Finally, to estimate the contribution of EV‐like nanoparticles and their component ferulic acid to lipid efflux in feces (Figure 4G), lipase activity was measured in the gastrointestinal contents of mice. An increase in lipase activity in duodenal fluids was observed in mice fed an HF/HS diet (Figure 5E and F). Administration of EV‐like nanoparticle–enriched fractions (PES‐ or PTFE‐filtered) as drinking water lowered the activity. A similar inhibitory effect on lipase activity was observed in the cecal contents. Ferulic acid was detected in duodenal fluids in mice that consumed EV‐like nanoparticle–enriched fractions, but not in the control group (Figure S8E).

Considering the differences in fecal lipid contents (Figure 4G), lipase activity between the two EV‐like nanoparticle–enriched fractions (PES filter‐treated and PTFE filter‐treated) shown in Figures 5A and F, and their respective ferulic acid concentrations (Figures 5C and S8E), the variation in lipase inhibitory activity can be partially attributed to the ferulic acid content, particularly the differences observed between groups administered PES filter‐treated and PTFE filter‐treated fractions. However, the results also suggest the involvement of additional factor(s) contributing to lipase inhibition in both filter‐treated fractions.

## 4 Discussion

We report that the administration of FBP or its water‐soluble fraction to mice reduces body weight gain, accompanied by decreases in food consumption and lipid absorption in the digestive tract. These effects are likely due to the lipase inhibitory activity of EV‐like nanoparticles present in FBP.

Microorganisms positively and negatively modulate cellular activities in our bodies. Small compounds, enterotoxins, and membrane surface molecules, such as lipopolysaccharides, induce inflammation by acting on our immune systems (Díaz‐Garrido et al. 2021; Nam et al. 2020; Watanabe et al. 2024). On the other hand, metabolites from microorganisms, such as lactate, propionate, and acetic acid, induce immune tolerance to achieve symbiosis (Guzmán‐Escalera et al. 2024). Symbiotic bacteria and their associated flora serve as a defense system against infections caused by harmful microorganisms and viruses (Cong et al. 2023). This protective effect is believed to be attainable through the consumption of fermented foods, which may help explain the long‐standing human tradition of incorporating them into the diet.

Humans often show aversion to unfamiliar fermented foods, potentially due to their strong or acquired taste. The taste receptors on the tongue detect acids, specifically the H^+^ ions, through acid receptors (Gonzales et al. 2009). These receptors play a crucial role in detecting potentially spoiled foods. As a result, fermented foods may initially be perceived as spoiled when first tasted. This may explain the results where the mice consumed less food when it was mixed with FBP (Figure S3B). Although not statistically significant, a slight increase in serum GLP‐1 levels was observed in mice that consumed FBP‐containing food or water. This may also contribute to the reduction in food intake (Sandoval and Magnan 2015).

Interestingly, the beverage containing the water‐soluble fraction of FBP, with only 0.5% solid content, reduced weight gain in mice. Considering that the lipid content in FBP is only 0.001% (Shimada et al. 2004), it suggests that most lipids in FBP might be present as water‐soluble substances, possibly EV or EV‐like nanoparticles. In fact, the water‐soluble fraction was found to contain EV‐like nanoparticles approximately 100 nm in size (Figure 1). When mice were fed the EV‐like nanoparticle‐enriched fraction, it reduced body weight gain, although it was less effective compared to the water‐soluble fraction (see Figure S3A and Figure 2B for comparison). The effects were evident when mice were fed a HF/HS diet (see Figure 3A). Similar findings regarding the essential role of gut flora in obesity have been reported (He et al. 2023; Lu et al. 2024). In contrast, gut microbes have been shown to promote fat digestion and absorption, and germ‐free mice exhibit resistance to HF diet‐induced obesity (Martinez‐Guryn et al. 2018).

A HF diet has been reported to shorten the colon in mice (Xie et al. 2020). We expect that fat is rapidly absorbed in the small intestine, thereby reducing the amount reaching the cecum and colon, which leads to colonic atrophy. Moreover, reduced function of the digestive tract may lead to decreased levels of GLP‐1 and GLP‐2. GLP‐2 protects the colon not only from short bowel syndrome in humans (Pironi et al. 2024), but also from model pathologies in mice (Minden et al. 2024), such as dextran sulfate‐induced inflammatory bowel disease (Gu et al. 2018), which is accompanied by colon shortening. Moreover, GLP‐2 exhibits anti‐inflammatory activity in the digestive tract (Aykan et al. 2019).

qPCR analyses showed that the mRNA levels of FATP1 (SLC27A4), a long‐chain fatty acid transporter, and FFAR4/GPR120, a long‐chain fatty acid receptor, were upregulated in the ileal mucosa of mice fed a HF/HS diet. Wu et al. (2006) reported that deficiency of FATP1 accelerated obesity in mice fed a HF diet. However, the isoform predominantly expressed in the intestine is FATP4 (SLC27A4) (Stahl et al. 1999). The mRNA level of FATP4 was reduced by 1.5‐fold in mice that consumed the EV‐like nanoparticle‐enriched fraction of FBP. Therefore, it is difficult to discuss fatty acid metabolism based solely on changes in the mRNA expression of fatty acid transporters.

Similarly, the mRNA levels of FFAR4 were also up regulated by the EV‐like nanoparticle‐enriched fraction of FBP. Systemic deficiency of FFAR4 has been shown to accelerate obesity in mice fed a HF diet (Oh et al. 2010). However, intestine‐specific FFAR4 deficiency in mice exhibits the opposite phenotype, resistance to obesity (Yasuda et al. 2023). In addition, FFAR4 mRNA expression in the intestine is generally up regulated by HF feeding, although it was down regulated in our experiments using ileal mucosal mRNA. Considering the higher expression of FFAR4 mRNA in the jejunum compared to the ileum (Yasuda et al. 2023), it is likely that most fatty acids were absorbed in the duodenum and jejunum, not in the ileum, in our mouse model, which may also explain a cause of colon atrophy. The EV‐like nanoparticle‐enriched fraction of FBP may suppress fat absorption in the small intestine and activate compensatory responses, such as restoring colon length.

These findings suggest that although the FBP‐derived EV‐like nanoparticles are taken up by HEK293 cells, their primary activity may occur in the intestinal lumen due to their lipase inhibitory properties. Unsaturated long‐chain fatty acids, such as oleic acid and α‐linolenic acid, have been reported to inhibit pancreatic lipase activity (Li et al. 2022). Additionally, lipstatin, which is isolated from *Streptomyces toxytricini*, possesses an acylglycerol‐like structure and exhibits inhibitory activity against pancreatic lipase (Bai et al. 2014). Its derivative orlistat is marketed for the treatment of obesity. Therefore, it is possible that the EV‐like nanoparticle‐enriched fraction contains fatty acids responsible for the observed lipase inhibition.

The appropriateness of using PTFE membranes to reduce EV‐like nanoparticles remains debatable. However, if the primary action of EV‐like nanoparticles is lipase inhibitory activity, then the observed correlations between the quantity of EV‐like nanoparticles and subsequent effects, such as reduced weight gain, can be reasonably explained, despite the weak statistical significance. A candidate compound responsible for the lipase inhibition is ferulic acid. Ferulic acid has been shown to exert anti‐diabetic effects by inhibiting glucose metabolism as well as pancreatic lipase activity in vitro and in animal models (Salau et al. 2021). We were also able to reproduce the strong inhibitory effect of ferulic acid against pancreatic lipase in our in vitro assay. However, a discrepancy remained between the reduction in lipase activity and the ferulic acid content in the fraction pre‐filtered through a PTFE membrane. These results suggest that multiple factors may contribute to lipase inhibition in FBP and its EV‐like nanoparticle‐enriched fraction. Ferulic acid, which is likely derived from raw materials used for fermentation (Kumor and Pruthi 2014) or produced as a metabolite by lactic acid bacteria (Westfall et al. 2019), is a representative compound known to inhibit pancreatic lipase.

Impairments in fatty acid metabolism and resistance to weight gain under HF diet feeding have been reported in aquaporin‐1‐deficient mice (Ma et al. 2001). Since aquaporin‐1 is essential for lipid absorption in the gastrointestinal tract, these mice do not exhibit weight gain when predominantly fed an HF diet. As a complementary effect, high lipase activity is observed in the duodenal lumen fluid and feces of these mice. These lines of evidence, together with the present results, suggest that EV‐like nanoparticles in FBP may directly inhibit lipase activity rather than fatty acid absorption.

FBP is well received by consumers due to its benefits in promoting intestinal regulation and its antioxidant properties (Shimada et al. 2004; Yang et al. 2015). An HF diet in mice and metabolic diseases in humans, such as diabetes, have been associated with constipation (Prasad and Abraham 2017; Taba Taba Vakili et al. 2015). Moreover, constipation is a common health concern across all age groups, particularly among the elderly population (Higgins and Johanson 2004). In this context, FBP may serve as an effective option for supporting gastrointestinal function through EV‐like nanoparticles, potentially via the promotion of GLP‐2 expression.

Further investigations are required to determine whether the EV‐like nanoparticles are capable of traversing the intestinal epithelium and entering the systemic circulation to exert their effects on distant organs, including the liver. Moreover, their intracellular functions following incorporation into target cells, as well as their potential roles in other gastrointestinal functions, warrant further elucidation.

## 5 Conclusion

FBP appears to reduce weight gain in mice fed high‐fat diets, likely via multiple mechanisms. Among these, the lipase inhibitory activity of EV‐like nanoparticles is particularly notable, contributing to weight reduction and improvement of hyperglycemia induced by consumption of an HF/HS diet. Colon atrophy observed in mice fed an HF/HS diet may be due to the rapid absorption of digested fat and sucrose in the proximal region of the intestinal tract, which results in reduced function in the distal region of the intestinal tract, including the colon. EV‐like nanoparticles in FBP may inhibit lipase activity in the intestinal tract, thereby reducing the rate of fat absorption and reactivating functions of the distal region of the intestinal tract, probably via enhanced levels of GLP‐2. Ferulic acid in EV‐like nanoparticle fractions may partially account for the lipase inhibitory activity.

## Author Contributions

**Kotomi Chikama**: investigation. **Koutarou Terada**: investigation. **Chika Yamamoto**: investigation. **Mana Yamamoto**: investigation. **Ayano Hojo**: formal analysis, investigation, methodology, validation. **Kotaro Fujioka**: project administration, formal analysis. **Hideto Torii**: project administration, formal analysis, validation. **Lee Wah Lim**: supervision, methodology. **Hiroshi Takemori**: supervision, writing–original draft, methodology, funding acquisition, formal analysis, project administration, validation.

## Conflicts of Interest

A.H., K.F., and H.Torii are employed by Manda Fermentation Co., Ltd. Other authors declare no conflicts of interest.

## Supporting information




**Supplementary Materials**: jfds70518‐sup‐0001‐SuppMat.pdf

## Data Availability

The data used to in this study are available from the corresponding author upon request.
