# Supplementary material for: Extracellular Vesicle‐Like Nanoparticles Present in Fermented Botanical Products Suppress Fat Absorption in the Gut
Source: J Food Sci. 2025 Aug 28;90(9):e70518. doi: 10.1111/1750-3841.70518 (PMC12395122; doi:10.1111/1750-3841.70518)
Supplement: Supplementary file 1 — Supplementary Materials: jfds70518‐sup‐0001‐SuppMat.pdf [file JFDS-90-0-s001.pdf]

Supplementary Table 1

Table S1 Sequences of primers used in quantitative PCR (qPCR) analyses.

Gapdh	mouse	5'-ACTCACGGCAAATTCAACGG
		5'-GACTCCACGACATACTGAGC
Gcg (Glp-1/2)	mouse	5'-GTGCAGTGGTTGATGAACAC
		5'-GCCTTTCACCAGCCAAGCAA
Dpp4	mouse	5'-GAGCTGTGAATCCAACTGTA
		5'-CATAGTCACAGATAGCCATCA
Abcg5	mouse	5'-CCAACCACAGGACTGGACTG
		5'-TCAGGACAGGGGTAACCACA
Abcg8	mouse	5'-AGAGTGGGCAACACGTATGT
		5'-CAGGTCAAATAGCCTGAAGA
Slc2a2 (Glut2)	mouse	5'- GGCTGCAAACAGCCTCTCAT
		5'- CCTGAGTGTGGTTGGAGCGAT
Ffar4 (Gpr120)	mouse	5'-GCCACCTGCTCTTCTACGTGA
		5'-CGGTTGGGCCAATCCAATGT
Slc27a1 (Fatp1)	mouse	5'-GACACCTACTCCAATGCTGT
		5'-GCTCCCGCCTCAGGTTGACA
Slc5a1 (Sglt1)	mouse	5'-GTGACGATGCCGGAGTATCT
		5'-GCGGTCTGCAGGGTATCTGT

The results of the transcriptome assay are available at:
https://www.researchgate.net/profile/Hiroshi-Takemori/publication/389993284_FBP_array1/data/67dc05f872f7f37c3e74ef5f/FBP-array1.xlsx?origin=profileOverview&_tp=eyJjb250ZXh0Ijp7InBhZ2UiOiJwcm9maWxlIiwicHJldmlvdXNQYWdlIjpudWxsLCJzdWJQYWdlIjpudWxsLCJwb3NpdGlvbiI6InBhZ2VDb250ZW50In19

Supplementary Figure 1

MANDA FERMENTATION CO., LTD.
5800-95 Innoshima Shigei-cho, Onomichi, Hiroshima, 722-2192 JAPAN
PHONE:(+81)-8452-4-3555　FAX:(+81)-8452-6-2034

Certificate of Analysis (COA)

1. Lot No.	P 1268
2. Result of Examination（試験結果）	
Standard Plate Count（一般生菌数）	less than 300 cfu/g
Coliform group（大腸菌群）	negative
Yeast and Mold（酵母、黴）	negative

5. Facility
MANDA FERMENTATION CO., LTD.（万田発酵株式会社　品質保証部）

MANDA FERMENTATION CO., LTD.

Akira Kato

Akira KATO
General Manager, Quality Assurance Dept.

Fig. S1 A copy of the certificate of absence of bacterial contamination in FBP.

Supplementary Figure 2

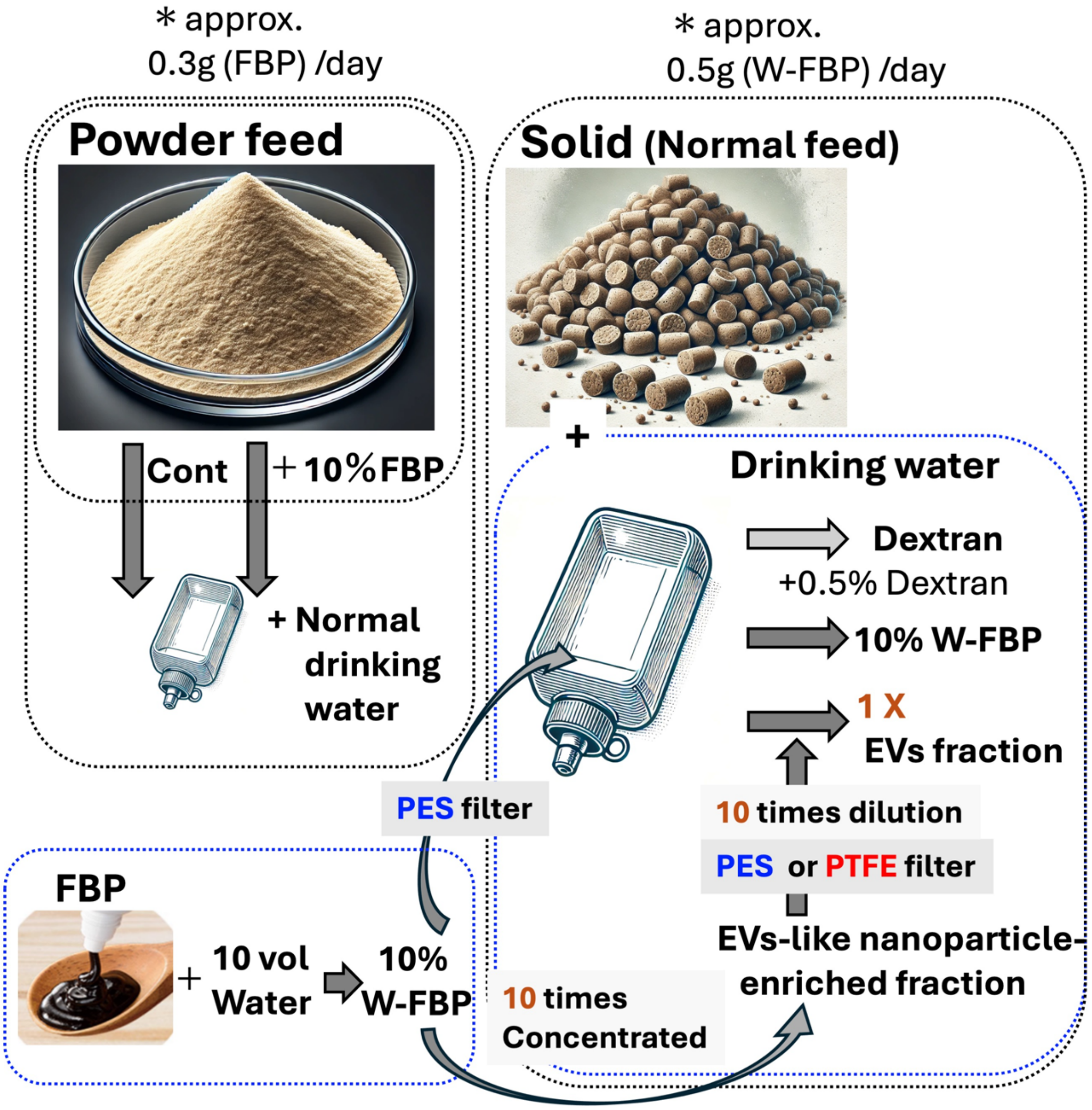

Fig. S2 Methods for the administration of FBP, the water-soluble FBP fraction (W-FBP), and the EVs-like nanoparticles-enriched fraction.

FBP was suspended in water at 10% (w/v), and the insoluble fraction was removed by centrifugation at 8000 rpm for 1 hour. The resulting supernatant was sterilized using a 0.45 μm polyether sulfone (PES) filter and administered as drinking water. Water containing 0.5% dextran was used as a control, as the solids content of the 10% water-soluble fraction of FBP was approximately 0.5%. Both feed and drinking water were replaced every two days. The EVs-like nanoparticles-enriched fraction was referred to as "1X" because it was recovered as a 10-fold concentrated solution and then diluted 10-fold with water. A polytetrafluoroethylene (PTFE) filter was also used to sterilize the EVs-like nanoparticles-enriched fraction. Given that mice consume approximately 3 g of feed and 5 mL of water per day, a 10% concentration corresponds to 0.3 g and 0.5 g of FBP.

Supplementary Figure 3

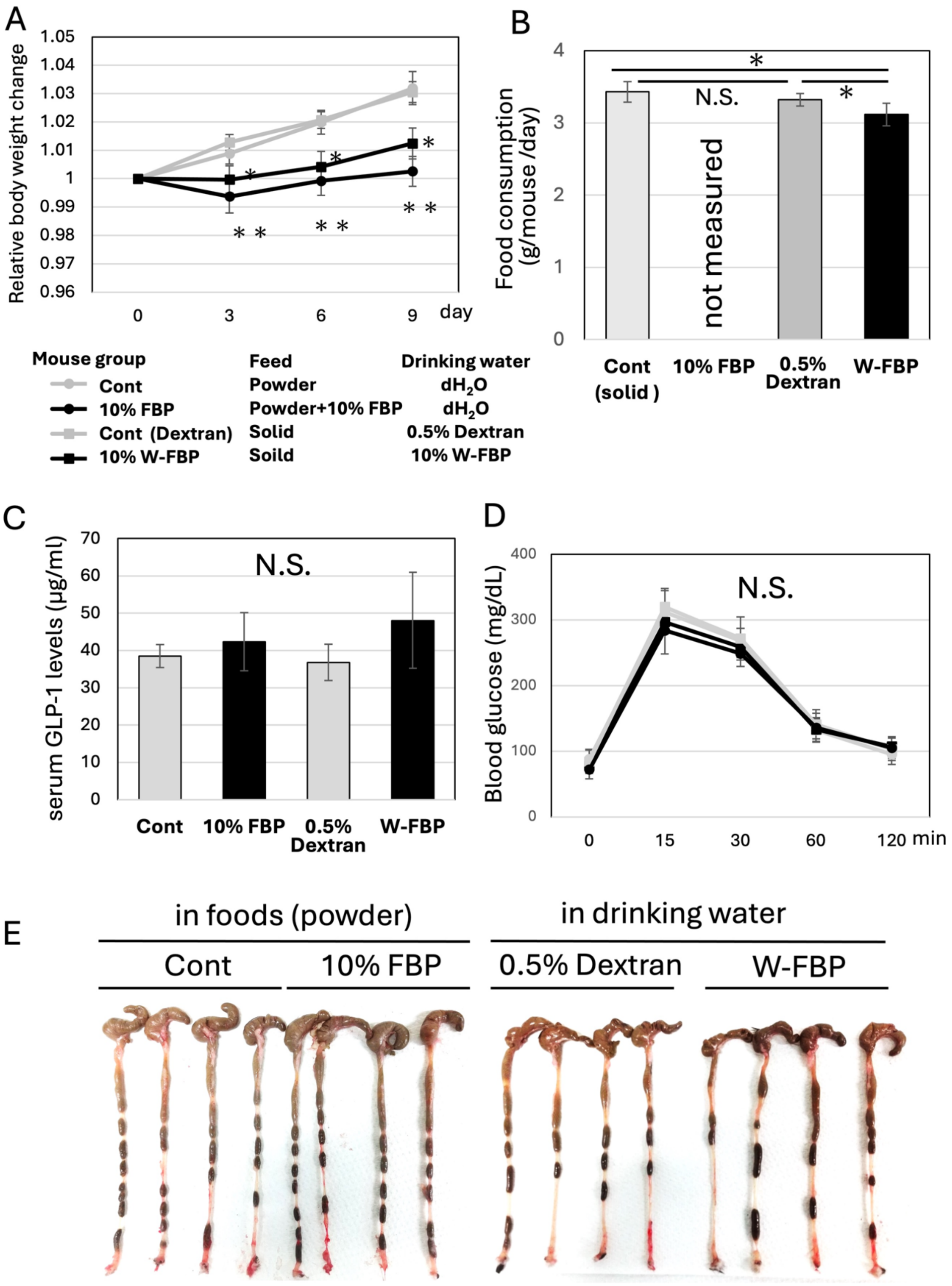

Fig. S3 A. Four group of mice with different feeds and drinking water were prepared. FBP (10% w/w) was mixed into powdered food (Cont and 10%FBP) or provided as 10% of a water-soluble fraction of FBP (W-FBP) in distilled water was served as in drinking water with with solid food (See Fig. S1). For the W-FBP control, 0.5% dextran-containing water was served. (see below) $n=8$. Means and S.D. are shown. *: $p<0.05$, **: $p<0.01$. B. Food consumption was monitored from the 4th to the 8th day. The group that consumed solid food with normal water served as the control. C. Serum total GLP-1 levels were measured on the final day. N.S., not significant. D. On the 8th day, mice underwent a 3-hour fasting followed by a glucose tolerance test (1g glucose via oral). E. Images of the colons were shown.

Supplementary Figure 4

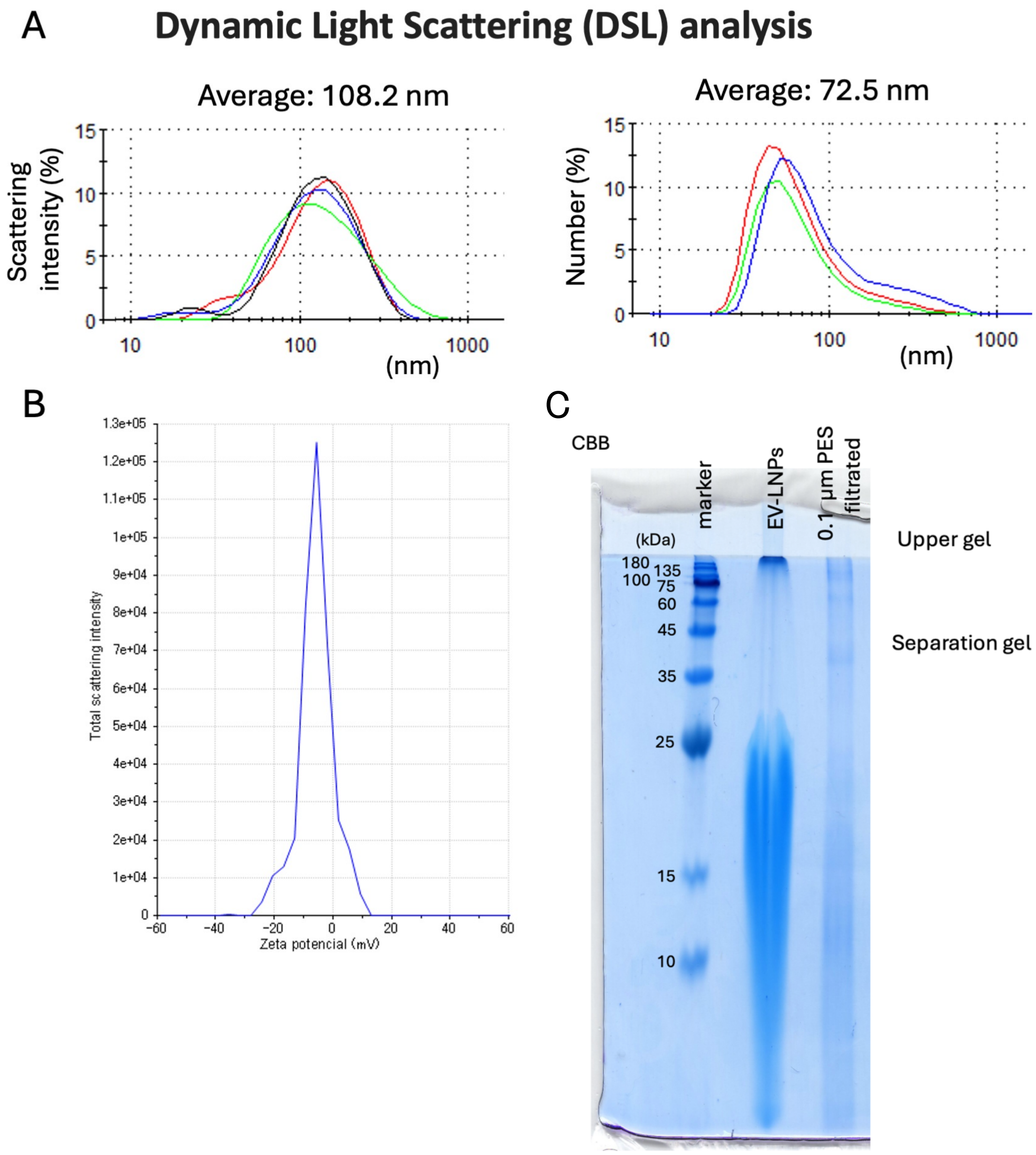

D

>180 kDa aggregates

K2C1_HUMAN **Mass:** 65999 **Score:** 64 **Matches:** 3(0) **Sequences:** 3(0)
Keratin, type II cytoskeletal 1 OS=Homo sapiens OX=9606 GN=KRT1 PE=1 SV=6

Query	Observed	Mr(expt)	Mr(calc)	Delta	Miss	Score	Expect	Rank	Unique	Peptide
130	487.2618	972.5091	972.5240	-0.0149	0	30	1.2	2	U	K.IEISELNR.V
164	533.2540	1064.4935	1064.5138	-0.0203	0	20	10	1	U	K.AQYEDIAQK.S
279	465.2327	1392.6764	1392.7249	-0.0485	1	14	41	1	U	R.TNAENEFVTIKK.D

Fig. S4 A. The average size of EVs-like nanoparticles of FBP was evaluated using Zetasizer Nano (for dynamic light scattering: DLS). Based on the average size obtained by scattering intensity and number, the values were 108.2 nm and 72.5 nm, respectively. B. The zeta potential of EVs-like nanoparticles was measured, and the results are summarized in Figure 1F. C. Original SDS-PAGE gel image for Figure 1G. Aggregated products (>180 kDa) were observed between the stacking gel and the separating gel, even after denaturation at 100 °C for 10 min in SDS sample buffer containing 10% mercaptoethanol. Neither small-molecule peptides nor the aggregated products were detected as major components in the 0.1 μm PES-filtered fraction (non-captured fraction). D. Proteome analysis detected only a contaminant protein (human keratin type 2) when the aggregated products were used as the sample. Keratin type 2 may have been detected as a contaminant during gel manipulation, as its expected molecular weight is approximately 65 kDa, not >180 kDa.

Supplementary Figure 5

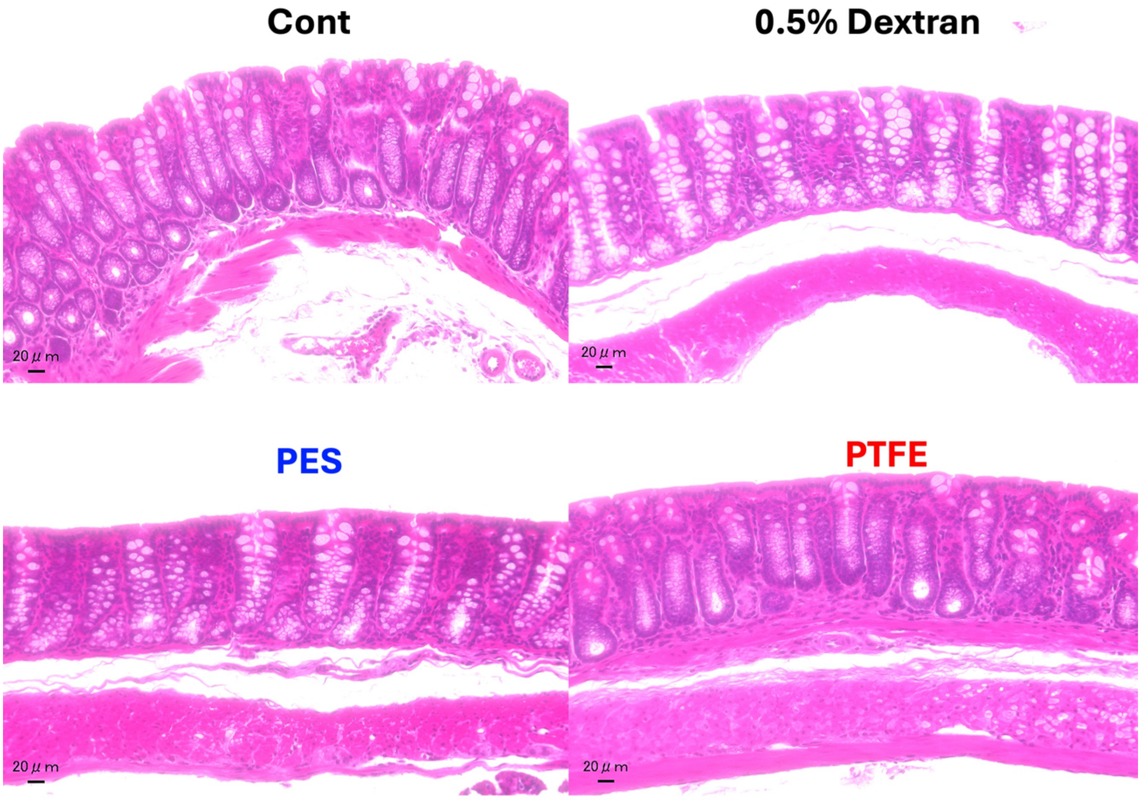

Fig. S5 Colons from Fig. 2E were sectioned and subjected to Hematoxylin and Eosin staining.

Supplementary Figure 6

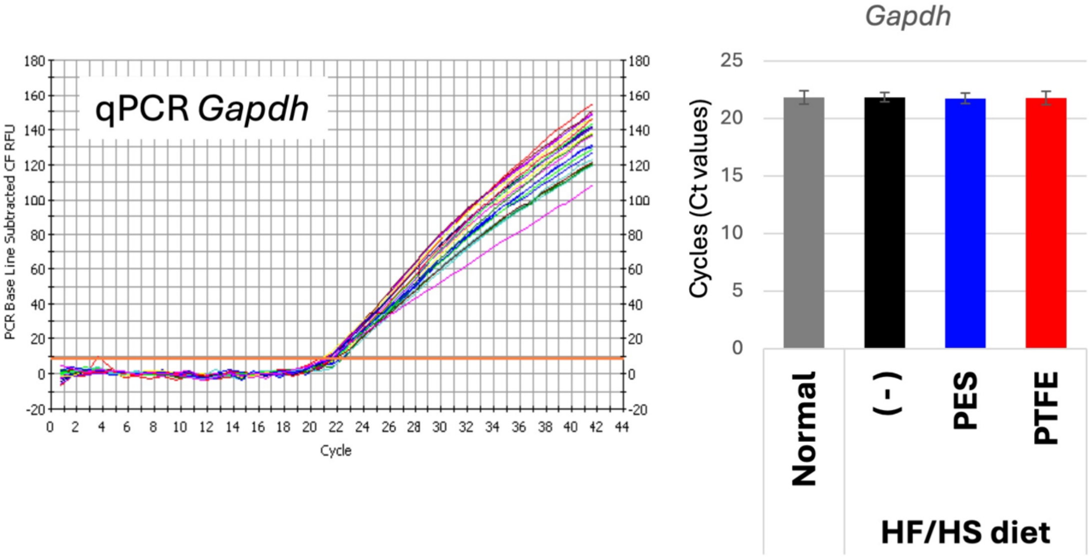

Fig. S6 A representative set of qPCR amplification curves for *Gapdh* mRNA is shown (*left*). No significant differences were observed among the experimental groups, as shown in Figure 3 (*right*).

Supplementary Figure 7

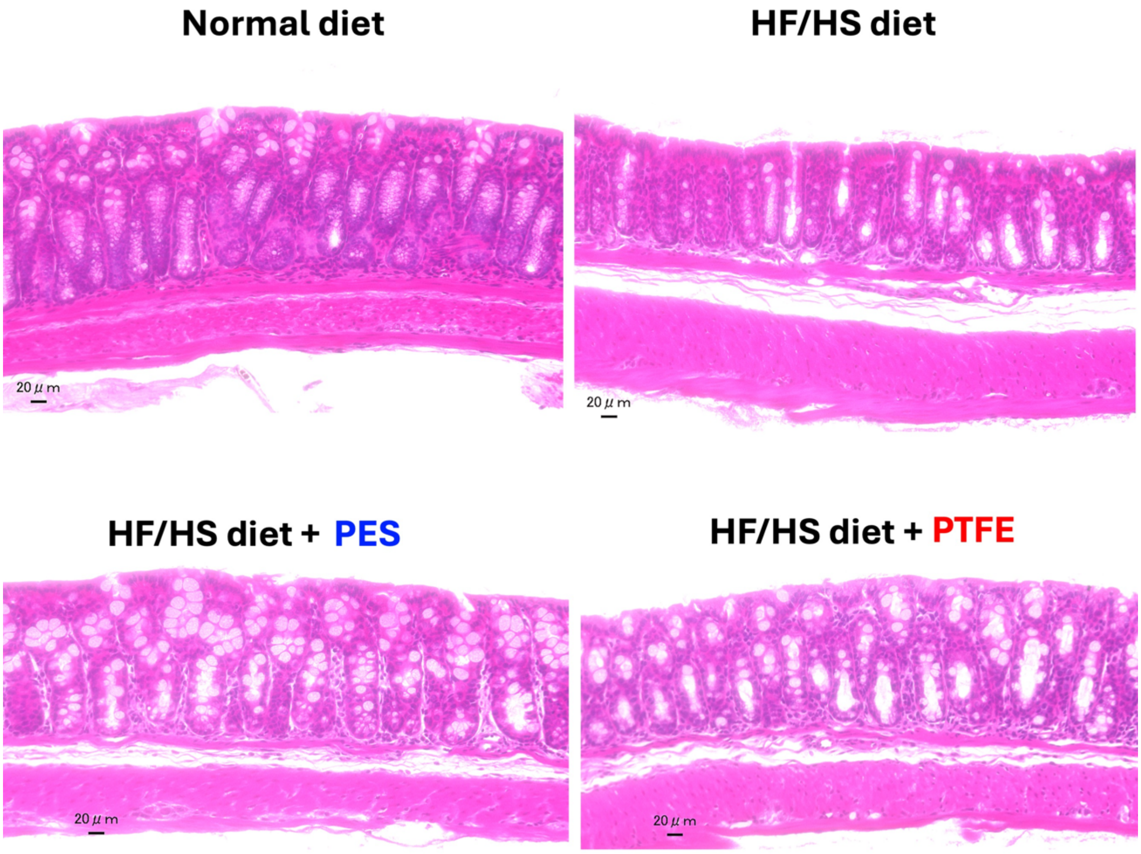

Fig. S7 Colons from Fig. 4A were sectioned and subjected to Hematoxylin and Eosin staining.

Supplementary Figure 8

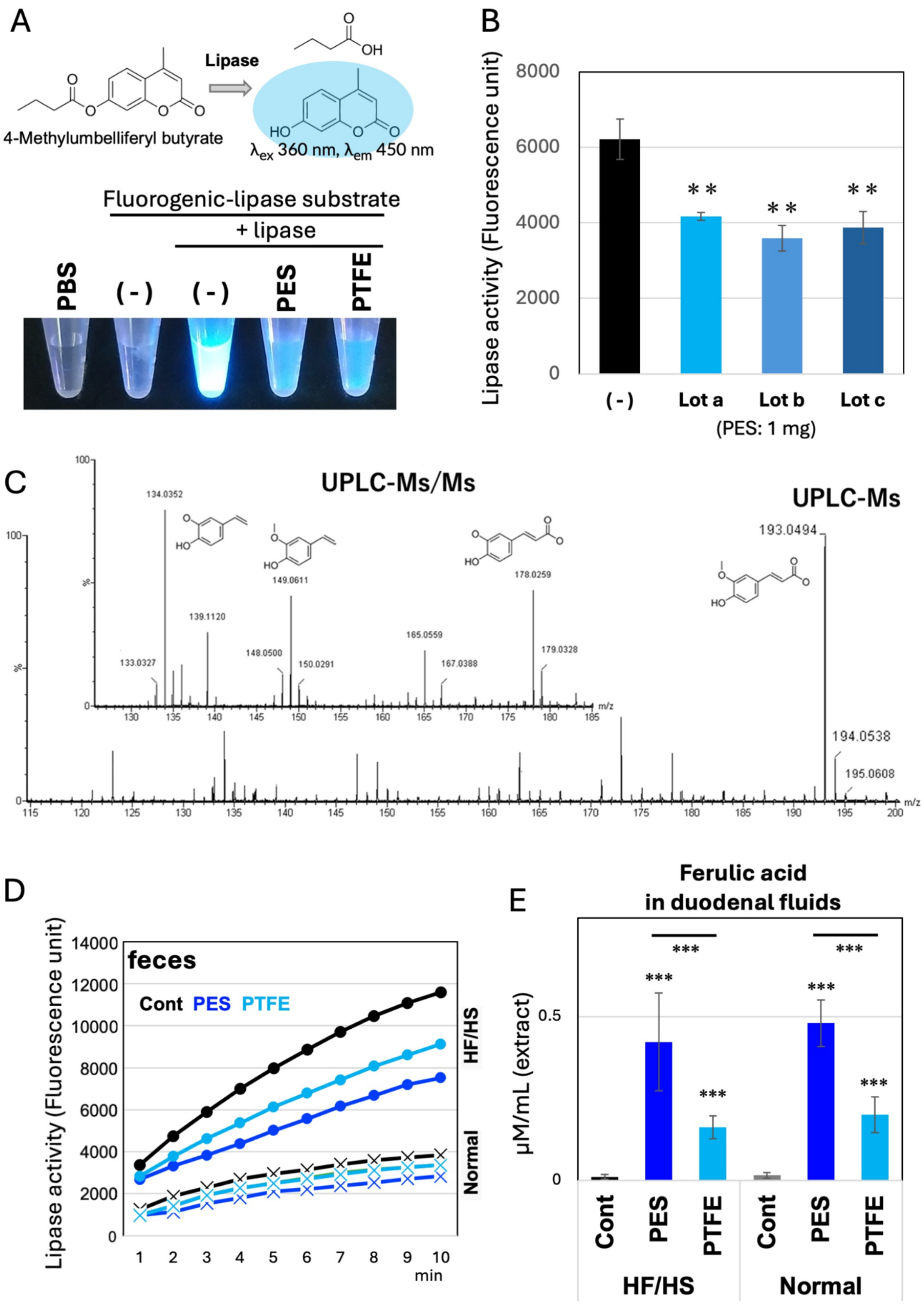

Fig. S8 A. Porcine pancreatic lipase（2 μg/0.2 mL）activity was measured using a fluorogenic lipase substrate (4-Methylumbelliferyl butyrate: 1 μM), which emits fluorescence after hydrolysis by lipase. EVs-like nanoparticle-enriched fractions (～5 μg lipids) were added to inhibit pancreatic lipase. An image was taken under a black light (Ex: 365 nm). B. Lot-to-lot variations in EVs-like nanoparticle-enriched fractions (1 mg lipids) derived from different FBP batches were assessed based on lipase activity ($n = 3$). C. UPLC-MS analysis of the 6.3 min peak (Figure 5B) was performed. The MS/MS spectrum and expected fragments are shown in the upper left corner. D. Lipase activity in feces collected from the cecum of the mice used in Figure 5E was measured. Mean values ($n = 4$) are shown. Refer to Figure 5F for variation and statistical significance. E. Ferulic acid in duodenal fluids used in Figure 5E ($n = 4$) was measured by HPLC using a C18 column (Figure 5C). Ferulic acid levels in control samples were below the detectable limit.
